# Busulfan exposure variability and acute toxicity in pediatric patients undergoing hematopoietic stem cell gene therapy

**DOI:** 10.3389/fphar.2026.1872108

**Published:** 2026-07-24

**Authors:** Daniele Canarutto, Simona De Gregori, Salvatore Recupero, Francesca Tucci, Francesca Fumagalli, Vera Gallo, Francesca Ferrua, Federica Barzaghi, Maddalena Migliavacca, Giulia Consiglieri, Matilde Cossutta, Matteo Doglio, Sabina Cenciarelli, Alessandro Nonis, Marcella Facchini, Alessandra Clerici, Raisa Jofra Hernandez, Sara Locatelli, Elena Albertazzi, Antonella Bartoli, Maria Pia Cicalese, Maria Ester Bernardo, Alessandro Aiuti, Valeria Calbi

**Affiliations:** 1 Faculty of Medicine and Surgery, Vita-Salute San Raffaele University, Milan, Italy; 2 San Raffaele Telethon Institute for Gene Therapy (SR-TIGET), Milan, Italy; 3 Pediatric Immunohematology Unit and BMT Program, IRCCS Ospedale San Raffaele, Milan, Italy; 4 Diagnostic Medicine Department: Clinical Chemistry Laboratory, Fondazione IRCCS Policlinico San Matteo, Pavia, Italy; 5 Department of Biomedicine and Prevention, PhD Program In Immunology, Molecular Medicine and Applied Biotechnologies, University of Rome Tor Vergata, Rome, Italy

**Keywords:** AUC, busulfan, gene therapy, pharmacokinetics, real-world

## Abstract

Accurate prediction of busulfan exposure is critical to achieve target myeloablative exposure while minimizing toxicity in pediatric hematopoietic stem cell transplantation. Model-informed precision dosing is recommended for improved accuracy and flexibility; dose adjustments in clinical practice are frequently performed using linear proportionality between dose changes and area under the concentration–time curve (AUC), optionally accounting for decrease of busulfan clearance over time. We retrospectively analyzed 54 consecutive pediatric patients receiving once-daily intravenous busulfan with therapeutic drug monitoring to characterize the relationship between dose adjustments and AUC variations, and to explore determinants of acute toxicity. Correlation between dose adjustments and AUC variations was linear for dose changes up to 1.16-fold, but linearity underestimated AUC increases following larger dose escalations. A quadratic model better described the dose–AUC relationship, particularly for dose increases, and was supported by an inverse correlation between dose variation and clearance change. While cumulative AUC was consistently maintained within target ranges, higher peak single-dose AUCs were associated with increased acute toxicity, including longer duration of mucositis and greater parenteral nutrition requirements. Initial dosing at 80 mg/m^2^ was associated with lower peak AUCs and reduced need for subsequent dose corrections compared with 120 mg/m^2^, without differences in engraftment outcomes. These findings highlight the clinical relevance of non-linear dose–exposure relationships and peak exposure in busulfan-related toxicity.

## Introduction

Tailoring busulfan regimens and making dose adjustments in pediatric patients is a critical step in achieving the target area-under-the-curve (AUC) and maximizing the probability of event-free survival (Domingos et al., 2024; Bartelink et al., 2016). Owing to the narrow therapeutic window of busulfan and the marked inter- and intra-patient pharmacokinetic variability, therapeutic drug monitoring (TDM) is an essential component of conditioning regimens in children. Model-informed precision dosing (MIPD) (Shukla et al., 2020), ideally implemented through dedicated pharmacokinetic software, is currently recommended for busulfan TDM and has shown superior accuracy and flexibility compared with simplified approaches (Domingos et al., 2024). However, despite these recommendations, the implementation of full MIPD remains heterogeneous across centers, and in routine clinical practice dose adjustments are still frequently performed using simplified assumptions based on proportionality between dose and exposure. The historical formula used for manual busulfan dose adjustments assumed linear dependence between relative AUC change (QAUC) and dose changes (QD) with unitary coefficient (QAUC = QD, i.e., Dose_new_ = Dose_old_ * (AUC_target_/AUC_observed_)). It has also been reported that busulfan clearance tends to decrease after the first administration by around 10% (Bartelink et al., 2012). To partially account for this phenomenon, a correction factor (commonly approximated as 0.9) has been proposed (QAUC = QD/0.9, i.e., Dose_new_ = 0.9 * Dose_old_ * (AUC_target_/AUC_observed_)) to mitigate overcorrection when linear proportionality is applied. Importantly, this correction represents a simplified empirical approximation rather than a substitute for MIPD-based dosing (Domingos et al., 2024).

In this context, and acknowledging that MIPD remains the preferred approach whenever available, we retrospectively analyzed a historical cohort of pediatric patients undergoing myeloablative, once-daily busulfan-based conditioning to characterize the real-world relationship between dose adjustments and the resulting changes in AUC when simplified linear assumptions are applied. In addition, we compared observed exposure changes with those expected under a linear model also incorporating a clearance-reduction correction factor.

Finally, we explored whether, at comparable levels of cumulative busulfan exposure, peak AUC was associated with the occurrence of acute toxicity, with the aim of providing insights into exposure–toxicity relationships in pediatric patients undergoing hematopoietic stem cell gene transplantation.

## Results

We retrospectively analyzed a cohort of 54 consecutive pediatric patients who received four once-daily doses of busulfan at IRCCS Ospedale San Raffaele between 2014 and 2024, with a target myeloablative cumulative AUC of 80–85 mg*h/L as part of conditioning for hematopoietic stem cell transplantation (Table 1). Median age was 2.2 years (range 0.6–15.5 years) and 18 of 54 patients were female; the underlying indications for conditioning included mucopolysaccharidosis (MPS) type I or IV and metachromatic leukodystrophy (MLD). Four patients received busulfan for allogeneic bone marrow transplantation and 50 received it in the context of investigational or approved autologous hematopoietic stem cell gene therapy (Consiglieri et al., 2024; Fumagalli et al., 2022; Fumagalli et al., 2025). Busulfan was administered either in combination with fludarabine (n = 12) or as a single agent (n = 42). The initial dose (D_1_) was age-based: 120 mg/m^2^ in 26 patients older than 1 year (median age 5.8 years) and 80 mg/m^2^ in 28 patients aged 1 year or younger (median age 0.9 years).

**TABLE 1:** Patient characteristics.

Parameter	D_1_ = 80 mg/m^2^	D_1_ = 120 mg/m^2^	p
N	28	26	​
Gender, M/F	21/7	15/11	0.25 (Fisher)
Years of age, median (range)	0.9 (0.6–6.5)	5.1 (1–15)	<0.01 (Mann-Whitney)
Gene therapy/HSCT	26/2	24/2	ns (Fisher)
cAUC, median (mg*h/L)	79.9	80.0	<0.01 (Fisher)
Patients with cAUC in Bartelink range (%)	96	96	ns (Fisher)
Neutrophil engraftment, median (range)	29 (16–49)	37 (15–46)	0.41 (Mantel-Cox)
Platelet engraftment, median (range)	31 (12–109)	32 (14–87)	0.90 (Mantel-Cox)

**FIGURE 1:**
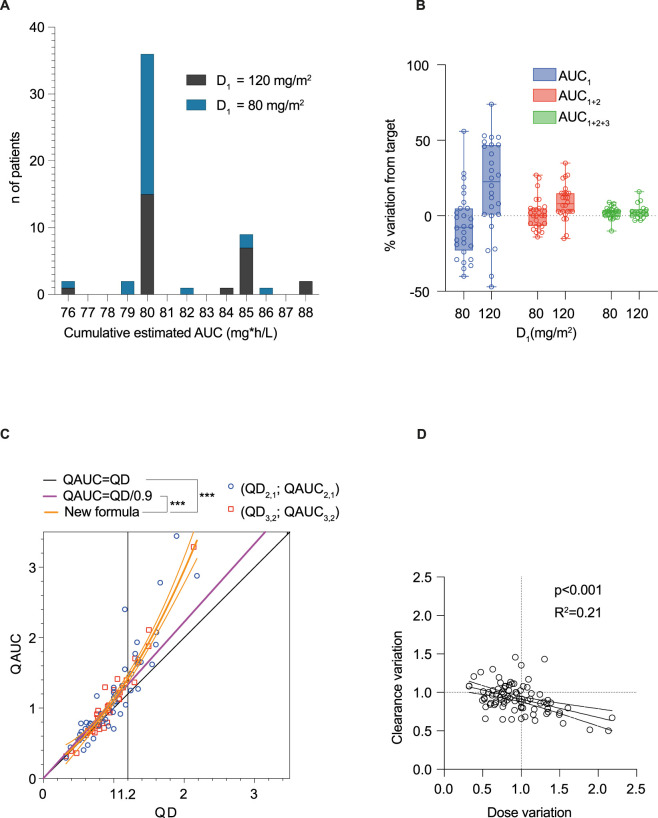
**(A)** Estimated cumulative AUC, assuming linear proportionality between dose adjustments and AUCs for non measured AUCs. **(B)** Variation between measured and target cumulative AUC for AUC_1_, AUC_1+2_, AUC_1+2+3_ stratified by dose 1 (D_1_). Bars indicate range. **(C)** Relative variations in busulfan dose (QD) and corresponding AUC variations (QAUC) with the standard correction formula (QAUC = QD, black), the EBMT formula (QAUC = QD/0.9, purple), and the new formula (QAUC = 0.11 + 0.59 QD + 0.42 QD^2^, orange) overlaid. Dotted lines indicate 95% confidence bands). Asterisks indicate p value for comparison of fits. Dose variations (QD) and corresponding AUC variations were calculated as follows: Dose variation (QD_n,n-1_) = Dose n (D_n_)/Dose preceding it (D_n-1_); AUC variation (QAUC _n,n-1_) = AUC n (AUC_n_)/AUC preceding it (AUC_n-1_); where n indicates either the 2^nd^ or 3^rd^ dose/AUC, and n-1 the preceding one. **(D)** Clearance variation upon busulfan dose variation (n = 85, line of best fit: y = −0.24x + 1.15; dotted lines indicate 95% confidence bands). *p < 0.05, **p < 0.01, ***p < 0.001, ****p < 0.0001, ns not significant.

AUC data were available for all patients for the first two doses (D_1_ and D_2_), and for 41 of 54 patients for D_3_. Cumulative AUC was estimated to be within the optimal 78–101 mg*h/L range (Bartelink et al., 2016) for 52 out of 54 patients (Figure 1A); after each dose, we calculated the deviation of the cumulative AUC from the corresponding target exposure at that time point. Deviations were greatest after the first dose, prior to dose adjustment, and were more pronounced in patients receiving an initial dose of 120 mg/m^2^ (Figure 1B).

We next evaluated the relationship between relative dose changes (QD) and relative AUC variation (QAUC) following dose adjustments using commonly used linear proportionality models, assuming either QAUC = QD or QAUC = QD/0.9, where the latter accounts for approximately 10% reduction in clearance. Both showed similar performance for modest dose changes (up approximately to QD ≤ 1.2), but failed to adequately describe observed data when busulfan dose increases exceeded ∼1.2-fold, where disproportionate non-linear increases in AUC were observed. Instead, the observed data were better explained by a quadratic model, Dose_new_ = Dose_old_ * $\frac{\sqrt[{2}]{0.16+1.68\left(\frac{AUCtarget}{AUCobserved}\right)}-0.59}{0.84}$ , (*R* ^2^ = 0.88), particularly for larger dose variations (Figure 1C). Comparison of model fits favored the quadratic model over both linear approaches (F test, p < 0.001 in both cases), even upon outlier removal (ROUT method, Q = 1%, p = 0.013, F test), and upon analysis of the two dose adjustments separately (QD_2,1_ and QD_3,2_; F-tests, both p < 0.05).

For more stringent validation of these findings, unconstrained by parametric coefficient assumptions and accounting for within-subject and between-subject variability, we then fitted and compared a linear mixed-effects model without the quadratic term (QAUC ∼ QD), against one fitted with the quadratic term (QAUC ∼ QD + QD^2^). The likelihood ratio test confirmed that the quadratic term significantly improved model fit (p = 0.0008); the quadratic model also had lower Akaike Information Criterion (AIC) values (−15.9 vs. −6.6), with progressive advantage over the linear one starting from QD > 1.16 in our dataset (Supplementary Figure S1).

Consistently with these findings, clearance variation showed a negative correlation with dose variation (Spearman r = −0.41, p < 0.001, n = 85, Figure 1D).

**FIGURE 2:**
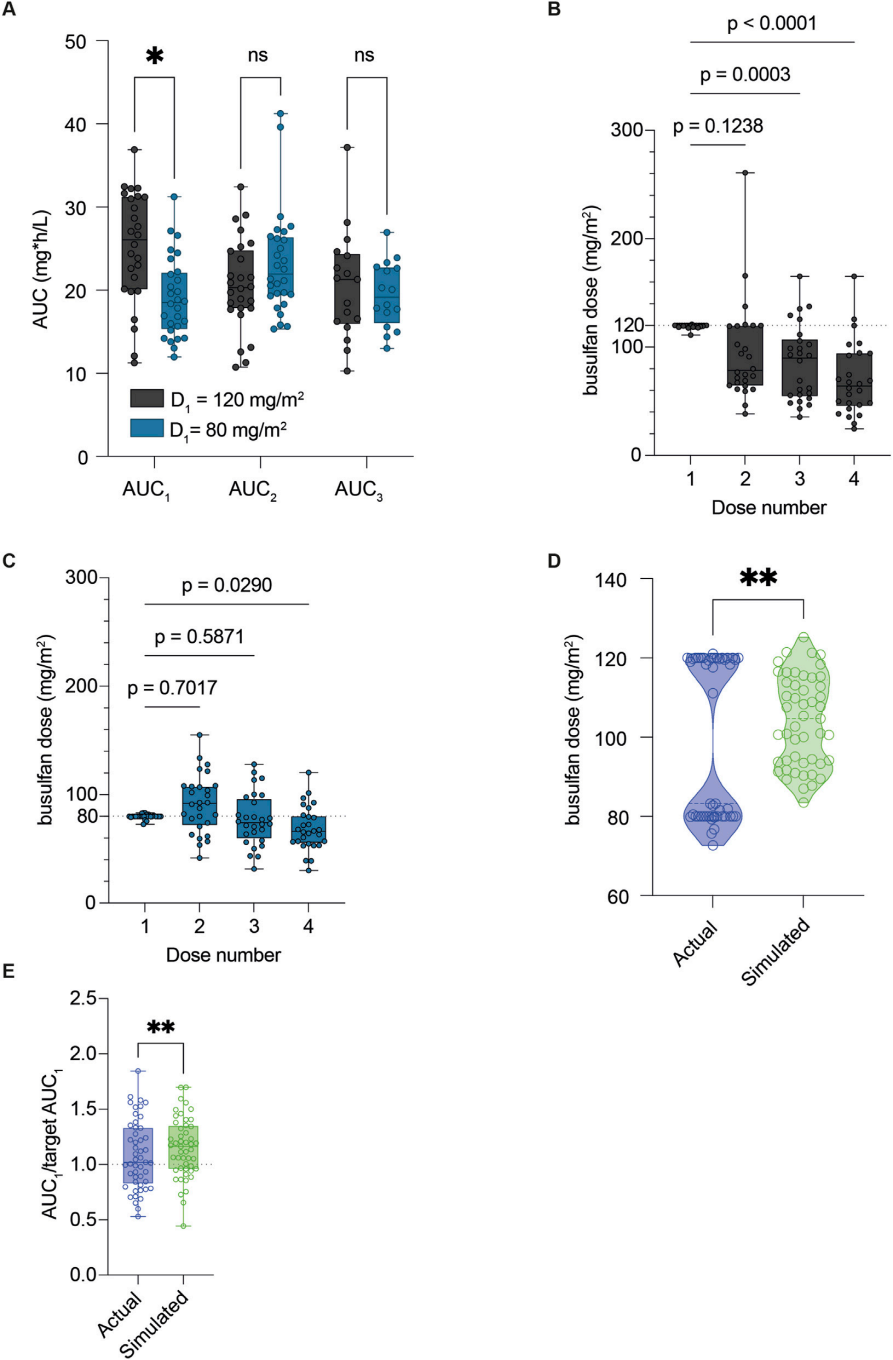
**(A)** AUC comparison following a first busulfan dose of 80 or 120 mg/m^2^. Multiple Mann-Whitney tests corrected for multiple comparisons. **(B,C)** Consecutive busulfan doses following administration of a first busulfan dose of 120 **(B)** or 80 mg/m^2^ **(C)**. Friedman test with Dunn’s multiple comparisons. **(E,F)** Actual and MIPD simulated (using Nexdose) busulfan D_1_ **(D)** and corresponding **(E)** AUC_1_, calculated assuming proportionality between model-informed proposed dose and actually administered dose, expressed as ratio over target AUC_1_. Wilcoxon matched-pairs signed rank test. *p < 0.05, **p < 0.01, ***p < 0.001, ****p < 0.0001, ns not significant.

Comparison of the two initial dosing regimens (120 vs. 80 mg/m^2^) showed that patients receiving 120 mg/m^2^ had a slightly higher cumulative AUC (80.04 vs. 79.99 mg*h/L, p < 0.01), a difference that was minimal in absolute terms and likely of negligible clinical impact. In contrast, these patients exhibited a significantly higher AUC after the first dose (AUC_1_) (Figure 2A) and greater intra-patient dose variability. Specifically, in patients receiving initial dose of 120 mg/m^2^ D_3_ and D_4_ were both lower than D_1_ (p < 0.01); instead, in the cohort of patients receiving an initial dose of 80 mg/m^2^ only dose 4 (D_4_) was significantly lower than D_1_ (p = 0.02, Figures 2B,C). Median maximum single-dose AUC was higher in patients receiving initial dose of 120 mg/m^2^ compared to those started at 80 mg/m2 (28.6 and 26.2 mg*h/L respectively, p < 0.01).

To account for age-related differences in busulfan metabolism, we also restricted the analysis to patients aged 1–7 years (n = 15 receiving a D_1_ of 120 mg/m^2^, median age 3.5 years and n = 12 receiving a D_1_ of 80 mg/m^2^, median age 1.9 years): differences in median AUC_1_ (Supplementary Figure S2) and median peak AUC remained statistically significant (28.2 vs. 24.9 mg*h/L, p < 0.05). We then performed a retrospective comparison of our nomogram-based choice of D_1_ with simulated first-dose exposure proposed by MIPD (by Nextdose). Simulated MIPD starting doses were higher than those actually administered (p < 0.01, Wilcoxon matched-pairs signed-rank test, Figure 2D) and would have resulted in significantly higher AUC_1_ values, potentially necessitating larger subsequent dose adjustments (p < 0.01, Wilcoxon matched-pairs signed-rank test, Figure 2E).

**FIGURE 3:**
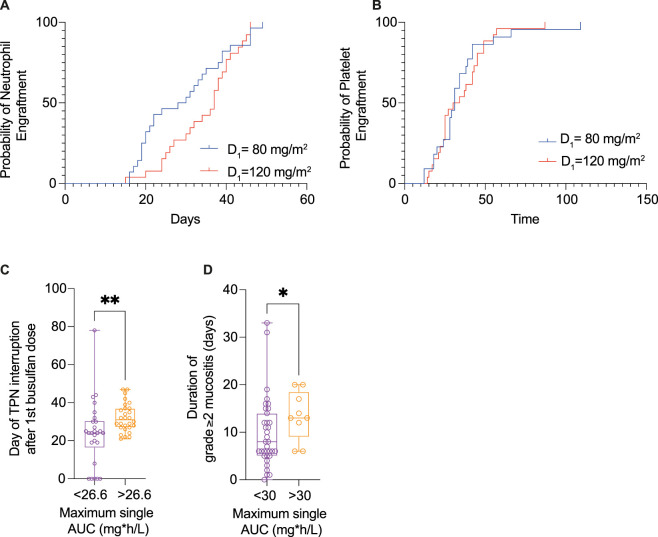
**(A)** Neutrophil engraftment, stratified by first busulfan dose. **(B)** Platelet engraftment, stratified by first busulfan dose. **(C)** Parenteral nutrition requirements in patients receiving it according to maximum single AUC exposure. Mann-Whitney test. n = 49. **(D)** Duration of grade≥2 mucositis according to maximum single AUC exposure. Mann-Whitney test. n = 44. *p < 0.05, **p < 0.01, ***p < 0.001, ****p < 0.0001, ns not significant.

We then assessed busulfan-related clinical outcomes. All patients achieved hematological engraftment. Neutrophil engraftment was achieved at median day 33 (range 15–49), and first of three consecutive days with platelets greater than 20,000/ul was day 31 (range 12–109). No significant differences in time to engraftment were observed between the two initial dosing groups (Figures 3A,B); peak AUC was not associated with time to neutrophil or platelet engraftment. Median follow-up was 5 years (range 0.26–10.9); three deaths unrelated to treatment occurred in patients with metachromatic leukodystrophy 0.65–1.1 years after gene therapy (GT), as previously reported (Fumagalli et al., 2022).

Receiving an initial busulfan dose of 120 mg/m^2^ was however associated with a higher likelihood of requiring Total Parenteral Nutrition (TPN) (odds ratio 12.4, p = 0.02), a surrogate indicator of mucositis severity. However, we identified by regression tree analysis peak AUC >26.6 mg*h/L as a stronger predictor than the initial dose itself for delayed discontinuation of TPN (+7 days, p = 0.0056 and p = 0.12 respectively, Figure 3C). Linear regression identified pathology (MLD vs. other) as significant predictor of TPN nutrition; restricting linear regression to MLD patients alone (n = 45) confirmed peak AUC >26.6 mg*h/L to be significantly associated with TPN duration (p = 0.02) irrespective of age (p = 0.95), gender (p = 0.13), and treatment (HSCT vs. GT).

Peak AUC >30 mg*h/L, i.e., a 50% overshoot from the 20 mg*h/L single dose target, was associated with longer duration of grade ≥2 mucositis (+7 days, p < 0.05, Mann-Whitney test, Figure 3D), yet this finding was not confirmed by linear regression accounting for covariates (p = 0.43). A trend towards earlier discontinuation of tramadol analgesia was also observed in patients receiving initial dose of 80 mg/m^2^ (−2 days, p = 0.06). In contrast total predicted cumulative AUC, which was similar in all patients as per conditioning design was not predictive of acute toxicity. Veno-occlusive disease occurred rarely (n = 3), with no statistical difference between groups likely reflecting the systematic use of prophylactic measures such as defibrotide. In summary, peak AUC, which is related to the initial D_1_, is correlated with direct and indirect measures of the severity of acute mucositis.

## Discussion

Achieving target AUC is a fundamental issue in HSCT owing to the narrow therapeutic index of busulfan, and pharmacokinetic (PK) variability. In this study, we provide real-world insights into the relationship between busulfan dose adjustments, exposure changes, and acute toxicity in pediatric patients with rare monogenic diseases undergoing myeloablative conditioning for hematopoietic-stem cell gene therapy or transplant.

Beyond confirming known busulfan pharmacokinetic principles, our study provides direct real-world evidence that dose escalation beyond approximately 1.2-fold is associated with a systematic deviation from linearity between dose and exposure, resulting in disproportionate increases in AUC despite mitigation strategies accounting for clearance reduction; note that our findings do not depend on how the dose was adjustment was calculated. Interestingly, another study in adult patients found that linearity between dose and exposure variations - with non-unitary coefficient - was reliable only up to the same 1.2-fold cutoff (Hall et al., 2026).

Consequently, assuming linear proportionality between dose and exposure can carry a substantial risk of overexposure when dose escalation exceeds modest thresholds, thus making adjustments following AUC underexposure particularly challenging. In our cohort, observed AUC changes upon dose adjustment were better described by a quadratic relationship, highlighting the limitations of simplified linear assumptions when applied to larger dose adjustments. Interestingly, reduction in busulfan clearance over time, reported in previous studies as a major determinant of intra-patient exposure variability especially in young children (Bartelink et al., 2012), was not confirmed in a more recent study (Mahomedradja et al., 2025). In our data, both variations in AUC and clearance were related to both the direction and magnitude of dose modifications, and this may partially accounting for such discrepancy, with potential implications for dose adjustment strategies.

The initial busulfan dose was determined in our cohort by nomogram, and not by MIPD, which is widely used to support individualized busulfan dosing (Shukla et al., 2020), recommended by the EBMT as good practice (Domingos et al., 2024). In our cohort, the target myeloablative AUC was reproducibly reached with both initial dosing regimens (80 mg/m^2^ and 120 mg/m^2^), with no relevant differences in engraftment kinetics. An initial dose of 80 mg/m^2^ was however associated with fewer subsequent dose adjustments and lower peak AUC values. These findings suggest that, when a fixed age-based nomogram is used instead of MIPD, a lower initial dose may offer a more favorable toxicity profile, even in older children without compromising engraftment. While this study was not designed to compare MIPD against empirical dosing, and no conclusions should be derived based on this data alone, in our retrospective simulations MIPD proposed starting doses were higher than those actually administered to patients and would have likely resulted in higher first-dose exposure (AUC_1_) (Figures 2D,E) with consequent higher dose adjustments. For the purpose of achieving targeted cumulative AUC, it is nevertheless recognized that neither MIPD nor nomogram-based choice of the initial dose is sufficient - without TDM - to achieve the target cumulative AUC (Domingos et al., 2024; Mahomedradja et al., 2025). Altogether, our findings are indeed consistent with the importance of intensive TDM, particularly when large dose modifications are required. While measurement of AUC after each dose is ideal, this is not always feasible in routine clinical practice. In such contexts, we highlight the risk that inaccurate exposure prediction following dose escalation may amplify the risk of excessive exposure.

We wish to stress that our findings should be interpreted as descriptive real-world behavior rather than as a proposal for an alternative dosing algorithm; they warrant independent validation and should not be taken as a clinical recommendation. Yet they can be informative for understanding exposure behavior in settings where full MIPD/PK models are not available; for supporting the interpretation of dose adjustments; and potentially for optimization of PK models.

In addition to cumulative exposure prediction, we found that, even within comparable cumulative AUC targets, peak AUC emerged as a relevant determinant of acute mucosal-related toxicity, including duration of mucositis and the need for total parenteral nutrition and analgesia. Importantly, our data show that, at clinically equivalent cumulative AUC targets, peak AUC is the key link between dose-adjustment to acute toxicity, underscoring the relevance of individual exposure trajectories beyond cumulative exposure alone. This observation highlights the importance of mitigating, when possible, the risk of high single-dose peaks, and underscores the clinical relevance of initial dose selection and early AUC assessment after substantial dose adjustments (Hughes et al., 2024). In a recent study comparing 3 h versus 6 h busulfan infusion duration in children and young adults maximum busulfan concentration was identified as independent predictor of overall survival and hepatotoxicity (Bae et al., 2026), independently supporting the notion that acute busulfan exposure is a potentially actionable determinant of toxicity. In the latter study maximum busulfan concentration was a better predictor than mean AUC; it remains to be determined whether peak AUC is a better or worse predictor than maximum busulfan concentration.

Several limitations of this study should be acknowledged. Its retrospective nature prevents establishing any causal relationship; the cohort was relatively homogeneous with respect to underlying disease and did not include patients transplanted for malignant indications, which may limit the generalizability of our findings to the broader transplant population. However, this relative homogeneity allows a clearer interpretation of busulfan pharmacokinetics. The predominant use of busulfan as a single conditioning agent reduces confounding effects related to prior intensive treatments, organ damage, or pharmacokinetic interactions with multi-agent conditioning regimens. AUC was calculated by non-compartmental analysis; upon recalculation of AUC_1_ by population PK modelling using Nextdose we confirmed however that the two are highly linearly correlated (*R* ^2^ = 0.97, p < 0.01), as reported (Hughes et al., 2024).

Initial dosing differed by age group; however, differences in AUC_1_ and peak AUC persisted when analyses were restricted to patients aged 1–7 years. Moreover, the higher toxicity observed in patients receiving higher initial doses supports the conclusion that early exposure intensity contributes meaningfully to acute toxicity. Prospective validation of these observations is warranted and will be the subject of future studies. Our study was not designed to directly compare the predictive accuracy of empirical dose adjustment versus PK-based models, which was hardly feasible due the combination of the different AUC estimation methods (non-compartmental vs. PK-based) and formulae used to suggest dose adjustments.

## Methods

### Study design and patient population

This was a retrospective, single-center observational study including consecutive pediatric patients who received myeloablative busulfan-based conditioning at IRCCS Ospedale San Raffaele between January 2014 and December 2024. Eligible patients were aged <18 years at the time of treatment and received four once-daily doses of intravenous busulfan as part of conditioning for hematopoietic stem cell transplantation. Patients were treated either in the context of allogeneic bone marrow transplantation or investigational/approved autologous hematopoietic stem cell gene therapy for inborn errors of metabolism. The study was conducted in accordance with institutional policies and the Declaration of Helsinki. Given the retrospective nature of the analysis, specific informed consent for this study was waived. Legal guardians provided written informed consent according to Italian law.

### Conditioning regimen and busulfan administration

Busulfan was administered intravenously once daily for four consecutive days. The target cumulative myeloablative AUC was 80–85 mg*h/L. Busulfan was given either as a single conditioning agent or in combination with fludarabine, according to disease-specific protocols (Figure 1A). This target range was chosen for being in the lower range of the Bartelink 79–101 mg*h/L range (Bartelink et al., 2016), being of documented effectiveness for the treatment of these diseases (Consiglieri et al., 2024; Fumagalli et al., 2022; Fumagalli et al., 2025). The initial busulfan dose (D_1_) was selected using an age-based nomogram, with doses of 80 mg/m^2^ or 120 mg/m^2^ (Bartelink et al., 2008). Subsequent doses (D_2_–D_4_) were adjusted based on TDM to achieve the target cumulative exposure.

### Therapeutic drug monitoring and AUC estimation

For each AUC, blood samples were collected shortly before infusion, and 5 min, 2 h, 4 h, 6 h after the end of the infusion, preferably from a peripheral venous access; busulfan concentration was measured according to institutional standard operating procedures using a validated HPLC-MS/MS analytical method, whose performance and accuracy parameters were validated according to EMA guidelines (Committee for Medicinal Products for Human Use CHMP, 2011). Individual AUC values were estimated using trapezoidal non-compartmental analysis; pharmacokinetic parameters were analysed by WinNonLin (Version 6.2.1, Pharsight-Phoenix) (Hughes et al., 2024). AUC was calculated after each available dose (AUC_1_–AUC_4_), and cumulative AUC was defined as the sum of individual daily AUCs and predicted AUCs (if not measured). Relative dose changes (QD) and relative AUC variations (QAUC) were calculated as the ratio of consecutive doses and corresponding AUCs, respectively.

In clinical practice, dose adjustments had been performed assuming linear dependence with unitary coefficient between QD and QAUC (Dose_new_ = Dose_old_ * (AUC_target_/AUC_observed_)). Cumulative AUC was estimated by adding together the measured AUCs (typically AUC_1_+AUC_2_+AUC_3_) to those estimated (AUC_4_ and sometimes AUC_3_).

### Simulation of model-informed precision dosing

Retrospective simulations of MIPD)–based starting doses were performed using a population pharmacokinetic framework. Simulated first-dose AUC (AUC_1_) was estimated assuming linear proportionality between dose and exposure. Nextdose (nextdose.org) was used to provide an estimate of model-based starting dose for n = 49 patients with complete data, and estimate clearance (Lawson et al., 2021). Due to intrinsic methodological differences between trapezoidal AUC estimation and population-based pharmacokinetic modeling, as well as between real-time dose adaptation and retrospective simulation, no comparison of predictive accuracy was possible.

### Assessment of clinical outcomes and toxicity

Acute toxicity endpoints included the occurrence and duration of grade ≥2 mucositis, requirement and duration of total parenteral nutrition (TPN), need for opioid analgesia, and incidence of veno-occlusive disease. Toxicities were graded according to CTCAE (https://dctd.cancer.gov/research/ctep-trials/for-sites/adverse-events/ctcae-v5-5x7.pdf). Engraftment was defined as the first of three consecutive days with an absolute neutrophil count >500/µL and platelet count >20,000/µL without transfusion support (Sureda et al., 2024).

### Statistical analysis

Comparisons between two independent groups were performed using the Mann–Whitney test. When multiple comparisons were performed across AUC measurements, p values were adjusted for multiple testing as indicated. Paired comparisons were performed using the paired t-test or Wilcoxon matched-pairs signed-rank test, as appropriate.

For comparisons of consecutive busulfan doses within the same individuals, repeated-measures analyses were conducted using the Friedman test with Dunn’s correction for multiple comparisons. Correlations between dose variation and clearance variation were assessed using Spearman’s rank correlation coefficient, and linear regression was used to describe the relationship between clearance variation and dose variation, with 95% confidence bands reported.

The equation Dose_new_ = Dose_old_ * $\frac{\sqrt[{2}]{0.16+1.68\left(\frac{AUCtarget}{AUCobserved}\right)}-0.59}{0.84}$ was calculated by quadratic fit on the entire dataset (QD_2,1_, QAUC_2,1_, QD_3,2_, QAUC_3,2_). Regression models describing the relationship between relative dose changes (QD) and relative AUC variations (QAUC) were compared using F tests. Outliers were identified and excluded using the ROUT method (Motulsky and Brown, 2006) (Q = 1%). Odds ratios for categorical clinical outcomes were estimated using logistic regression.

Linear and quadratic mixed-effects models were fitted to examine the relationship between QAUC and QD using the nlme package in R. Random intercepts were specified for subjects, and model comparisons were conducted using likelihood ratio tests and Akaike Information Criterion (AIC).

For identification of the best AUC cutoffs discriminating acute toxicity outcomes, a decision tree regression model was fitted and implemented in R using the rpart package specifying ANOVA method for continuous outcomes. Tree growth was constrained to a minimum node size of 8 observations.

The association between predictors and TPN days or mucositis duration was assessed using R. A linear regression model was fitted including the previously identified maximum AUC cutoff, sex, age, underlying disease and procedure (gene therapy or HSCT) as covariates.

All statistical analyses were performed using GraphPad Prism and R software. A two-sided p value < 0.05 was considered statistically significant.

## Data Availability

The datasets presented in this article are not readily available because of the small number of participants in the study and potential for identification, individual patient data beyond what is included in the manuscript will not be available. Requests to access the datasets should be directed to calbi.valeria@hsr.it.

## Supplementary material

SUPPLEMENTARY FIGURE S1 Identification of range of QD values for which non-linearity becomes relevant. A sequence of candidate cutoff values spanning the observed QD range was defined, and for each the dataset restricted to that range (with a minimal numerosity of 30). Within each set linear and quadratic mixed-effects models were fitted and the difference in AIC compared (ΔAIC = AIC quadratic model – AIC linear model).

SUPPLEMENTARY FIGURE S2 AUC comparison following a first busulfan dose of 80 or 120 mg/m2 for patients aged 1–7 years only. Multiple Mann-Whitney tests corrected for multiple comparisons.
