# Supplementary material for: Busulfan exposure variability and acute toxicity in pediatric patients undergoing hematopoietic stem cell gene therapy
Source: Front Pharmacol. 2026 Jul 24;17:1872108. doi: 10.3389/fphar.2026.1872108 (PMC13448675; doi:10.3389/fphar.2026.1872108)

AIC comparison

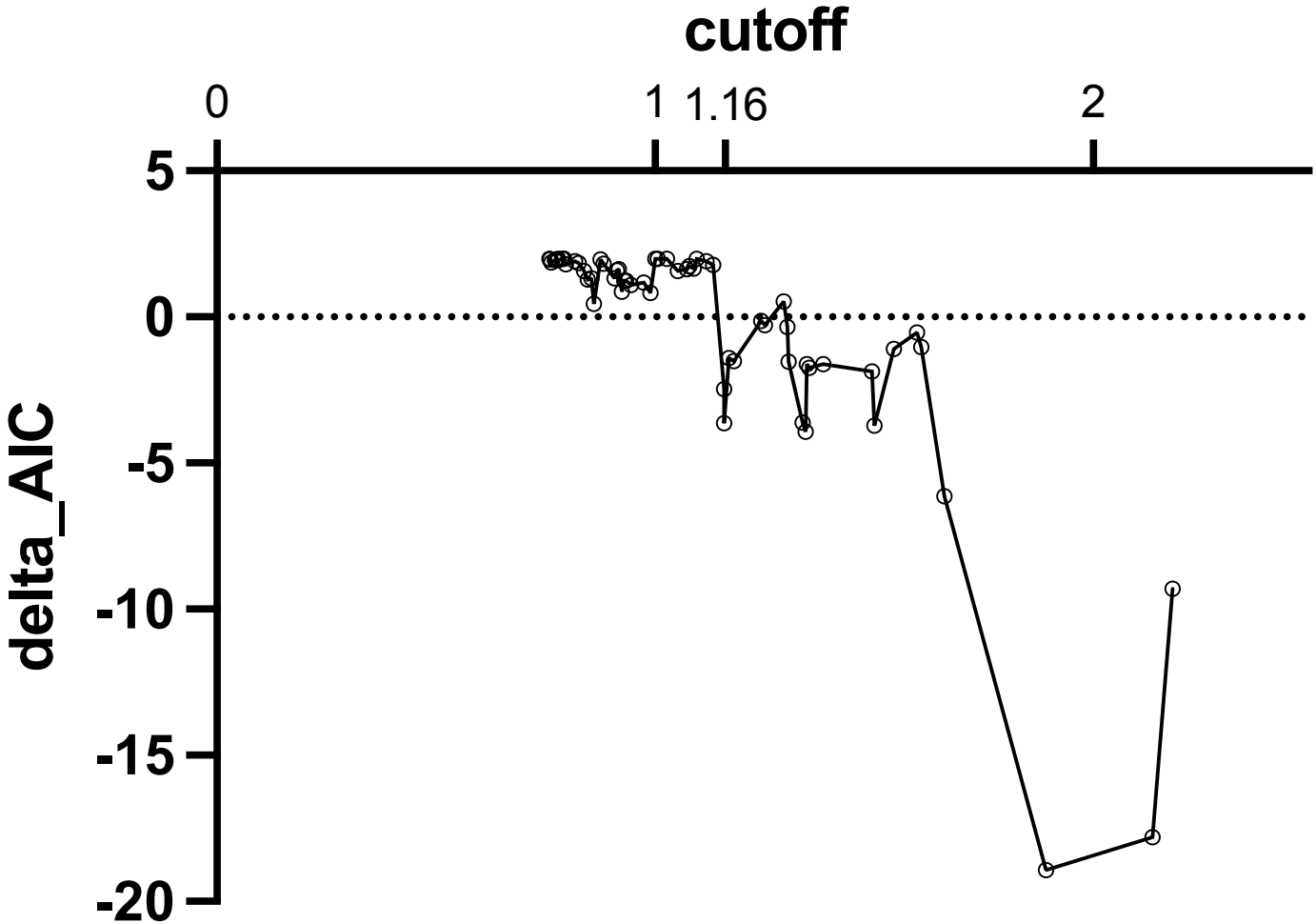

Supplement: Supplementary file 1 [file DataSheet1.pdf]
