# Supplementary material for: Busulfan exposure variability and acute toxicity in pediatric patients undergoing hematopoietic stem cell gene therapy
Source: Front Pharmacol. 2026 Jul 24;17:1872108. doi: 10.3389/fphar.2026.1872108 (PMC13448675; doi:10.3389/fphar.2026.1872108)
Supplement: Supplementary file 2 [file DataSheet2.pdf]

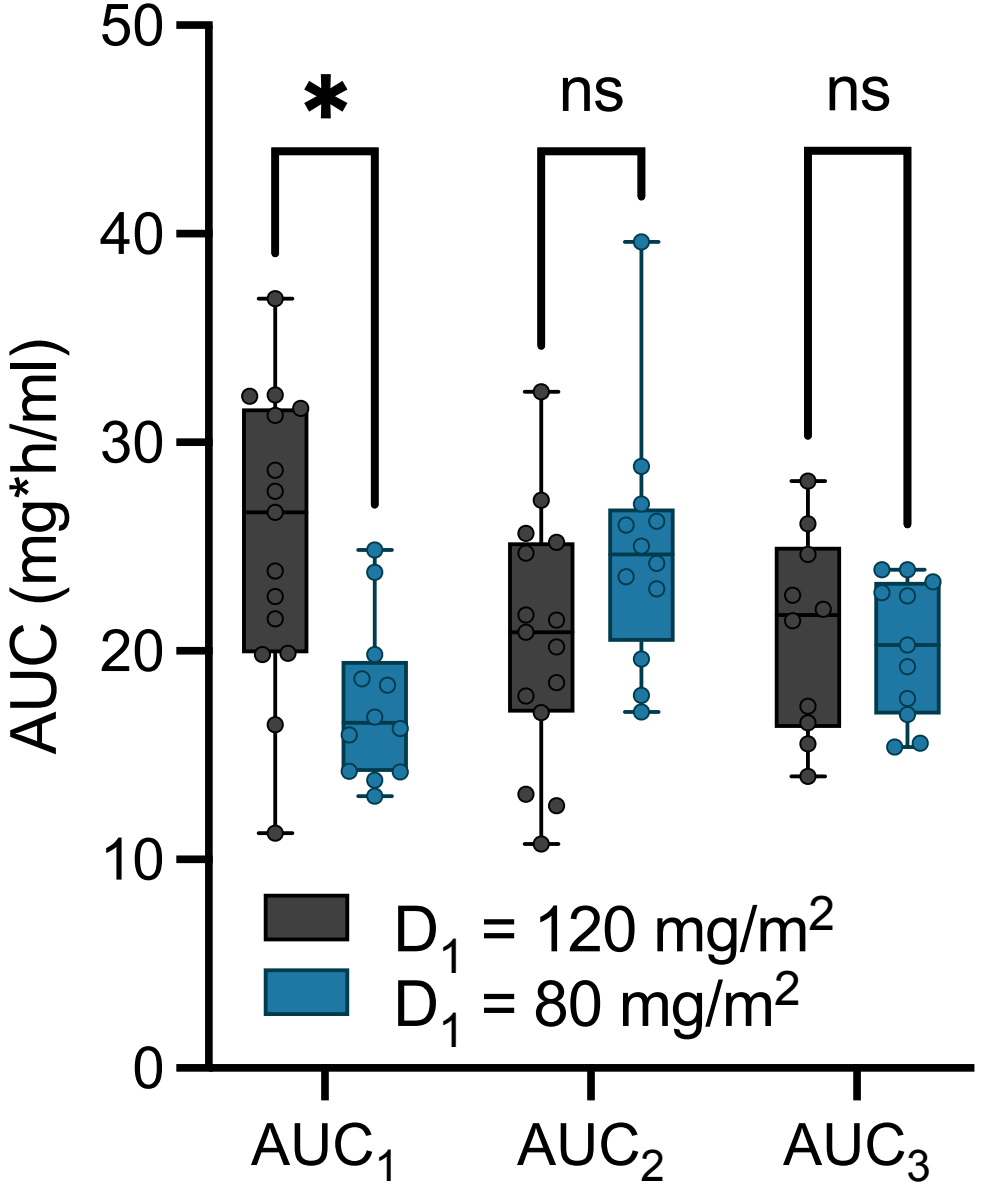

AUC (mg*h/ml)
50
40
30
20
10
0
*
ns
ns
D1 = 120 mg/m2
D1 = 80 mg/m2
AUC1
AUC2
AUC3
